# (F)uridylylated Peptides Linked to VPg1 of Foot-and- Mouth Disease Virus (FMDV): Design, Synthesis and X-Ray Crystallography of the Complexes with FMDV RNA-Dependent RNA Polymerase

**DOI:** 10.3390/molecules24132360

**Published:** 2019-06-26

**Authors:** Sonia de Castro, Cristina Ferrer-Orta, Alberto Mills, Gloria Fernández-Cureses, Federico Gago, Nuria Verdaguer, María-José Camarasa

**Affiliations:** 1 Instituto de Química Médica (IQM-CSIC), Juan de la Cierva 3, E-28006 Madrid, Spain; 2 Institut de Biologia Molecular de Barcelona (IBMB-CSIC), Parc Científic de Barcelona, Josep Samitier 1–5, 08028 Barcelona, Spain; 3 Area de Farmacología, Departamento de Ciencias Biomédicas, Unidad Asociada al IQM-CSIC, Universidad de Alcalá, E-28805 Alcalá de Henares, Madrid, Spain

**Keywords:** foot-and-mouth disease virus, RNA-dependent RNA polymerase, 5-fluorouracil, uridylylation inhibition, VPg

## Abstract

Foot-and-mouth disease virus (FMDV) is an RNA virus belonging to the *Picornaviridae* family that contains three small viral proteins (VPgs), named VPg1, VPg2 and VPg3, linked to the 5′-end of the viral genome. These VPg proteins act as primers for RNA replication, which is initiated by the consecutive binding of two UMP molecules to the hydroxyl group of Tyr3 in VPg. This process, termed uridylylation, is catalyzed by the viral RNA-dependent RNA polymerase named 3D^pol^. 5-Fluorouridine triphosphate (FUTP) is a potent competitive inhibitor of VPg uridylylation. Peptide analysis showed FUMP covalently linked to the Tyr3 of VPg. This fluorouridylylation prevents further incorporation of the second UMP residue. The molecular basis of how the incorporated FUMP blocks the incorporation of the second UMP is still unknown. To investigate the mechanism of inhibition of VPg uridylylation by FUMP, we have prepared a simplified 15-mer model of VPg1 containing FUMP and studied its x-ray crystal structure in complex with 3D^pol^. Unfortunately, the fluorouridylylated VPg1 was disordered and not visible in the electron density maps; however, the structure of 3D^pol^ in the presence of VPg1-FUMP showed an 8 Å movement of the β9-α11 loop of the polymerase towards the active site cavity relative to the complex of 3D^pol^ with VPg1-UMP. The conformational rearrangement of this loop preceding the 3D^pol^ B motif seems to block the access of the template nucleotide to the catalytic cavity. This result may be useful in the design of new antivirals against not only FMDV but also other picornaviruses, since all members of this family require the uridylylation of their VPg proteins to initiate the viral RNA synthesis.

## 1. Introduction

Foot-and-mouth disease (FMD), a highly contagious viral disease affecting cloven-hooved livestock as well as wildlife species, is caused by FMD virus (FMDV). FMD has a great economic impact on the global livestock industry and undermines food security [1,2]. According to the OIE (World Organization for Animal Health) report of 2017 [3], FMD is endemic in several regions of Asia, a great part of Africa and also Middle East countries. Moreover, the FMD-free countries (where the disease was previously eradicated) are under continuous threat of an incursion of FMD. In this case, outbreaks of the disease represent approximately 1.5 billion dollars per year in losses. In endemic regions, the cost is estimated to be >6.5 billion dollars per year [1]. Therefore, the control and reduction of the impact of FMD will have a highly positive economic effect on both FMD-free and FMD-infected countries.

FMDV is a member of the *Picornaviridae* family and its genome consists of a positive-sense single-stranded RNA molecule, of around 8 Kb, that has a small peptide linked to its 5′-end called viral protein genome-linked or VPg. VPg plays a key role in the initiation of genome replication, acting as a primer for RNA synthesis [4]. Replication of the FMDV genome is mediated by a viral RNA-dependent RNA polymerase (RdRp), named 3D^pol^ through a negative-sense RNA intermediate. RdRp uses VPg as a primer for both minus and plus strand RNA synthesis. The first step of the viral genome replication consists of the successive incorporation of two uridine-monophosphate residues to a highly conserved tyrosine residue (Tyr3) of VPg. This reaction, termed uridylylation, is also catalyzed by 3D^pol^ using as template a small stem-loop structure (cis-acting replication element (cre) or 3B-uridylylation site (bus)) that is located within the 5′-untranslated region [5,6,7]. Hence, the hydroxyl group of Tyr3 forms a phosphodiester bond with the first UTP molecule to form VPgpU; then, VPgpU “slides back” one base-pair and a second UTP is incorporated to form VPgpUpU, which acts as a protein primer for the synthesis of the genomic RNA.

**Figure 1:**
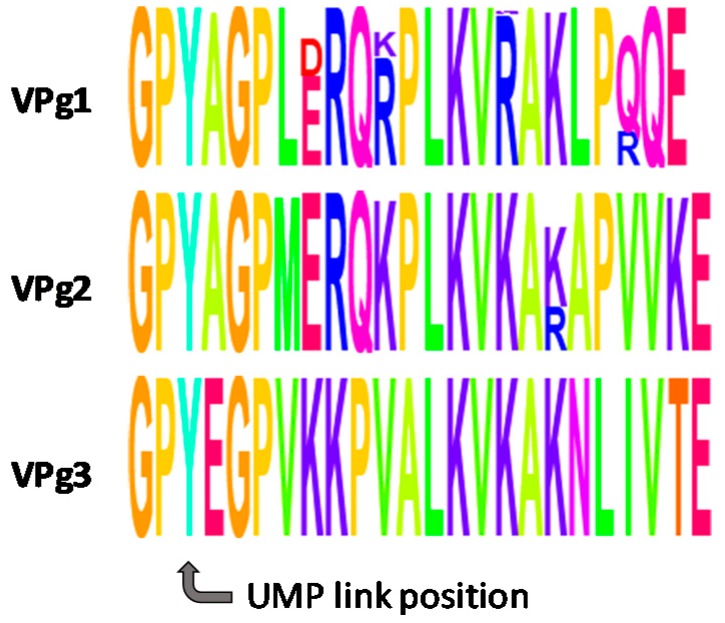
Amino acid sequence WebLogos of foot-and-mouth disease virus (FMDV) viral protein genome-linked (VPgs). The overall height of each stack indicates the sequence conservation at that position whereas the height of symbols within the stack reflects the relative frequency of the corresponding amino acid at that position.

Unlike other picornaviruses, which express a single VPg protein, the genome of FMDV encodes three similar but not identical VPg copies (VPg1, VPg2 and VPg3) [8] and all three of them are linked to the viral RNA [5,9]. These VPgs are 23 or 24 amino acids long, can be uridylylated (Figure 1) and are active as replication primers [10]. Although deletion as well as insertion of one VPg gene still results in infective particle production, optimal viral RNA synthesis and FMDV viability require all three copies [2,11,12].

The x-ray crystal structures of the complexes between FMDV 3D^pol^ and the uridylylated and non-uridylylated forms of VPg1 have been determined [12]. Both complexes showed that VPg1 is located inside the central cleft of the polymerase, positioning the hydroxyl group of Tyr3 to mimic the free 3′-hydroxyl group of a nucleic acid primer at the polymerase catalytic site [12]. Moreover, a combination of these crystal complexes with site-directed mutagenesis of 3D^pol^ and chemical synthesis of mutant VPg proteins, allowed the determination of the importance of several residues of 3D^pol^ for initiation of RNA synthesis [2,12]. The structure of the complexes showed the positioning of 15 out of the 23 amino acids of VPg1. It was also possible to trace one or two additional poorly ordered VPg amino acids, exiting from the polymerase central cavity while the remaining six C-terminal residues were completely disordered [12].

Despite the key role of 3D^pol^ in the life cycle of the FMDV, there are currently very few molecules described as inhibitors of the virus replication [13,14,15,16,17]. Among them, 5-fluorouridine triphosphate (FUTP) is a potent competitive inhibitor of VPg uridylylation in vitro [18] and also behaves as a potent mutagen for picornaviruses including FMDV [19,20,21,22]. The affinity of 3D^pol^ for this compound is 3–10 times higher than that of UTP depending on the experimental conditions. Mass spectrometry analysis of the in vitro uridylylation and fluorouridylylation products allowed the identification of VPg fragments containing UMP or FUMP covalently linked to the OH of Tyr3 of VPg. The fact that FUTP behaves as a competitive inhibitor of uridylylation indicates that both nucleotide triphosphates (UTP and FUTP) compete for the same uridylylation site (Tyr3) [12,15]. This fluorouridylylation prevents further incorporation of the second UMP residue to form the corresponding VPg-pFUpU and therefore initiation of RNA synthesis is inhibited. On the contrary, incorporation of FUMP into the nascent dsRNA by the polymerase, in place of UMP or CMP, does not prevent chain elongation. Moreover, in some conditions, it favors misincorporations at downstream positions, hence its potent mutagenic activity [18]. Both facts may explain the dual inhibitory and mutagenic properties of 5-fluorouracil (FU) against picornaviruses. However, the molecular basis of how FUMP incorporation blocks further UMP incorporation in VPg is still unknown. The elucidation of this mechanism of inhibition of VPg uridylylation by FUTP could be useful not only for FMDV, but also for the inhibition of other picornaviruses, since all of them use VPg proteins as primers.

**Figure 2:**
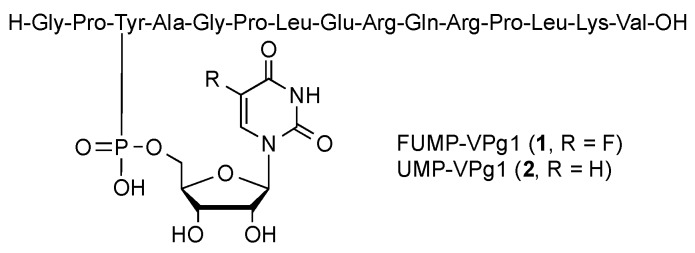
Fifteen-mer model of FUMP-VPg1 and UMP-VPg1.

In an attempt to propose a possible mechanism of inhibition of FMDV VPg uridylylation by FUMP, we synthesized a shortened version of FUMP-VPg1 (**1**, Figure 2), containing the first 15 amino acids of VPg1 that were unequivocally traced in the previous x-ray analyses [2], to study its structure in complex with 3D^pol^. For the purpose of comparing structural differences, we also decided to synthesize the non-fluorinated UMP-VPg1 molecule (**2**, Figure 2). The structural alterations in the 3D^pol^ active site on FUMP-VPg1 binding have been examined by comparing the three-dimensional structures of 3D^pol^ crystallized in the presence of either FUMP-VPg1 or UMP-VPg1 by x-ray crystallography.

## 2. Results and Discussion

### 2.1. Chemical Synthesis

**Scheme 1:**
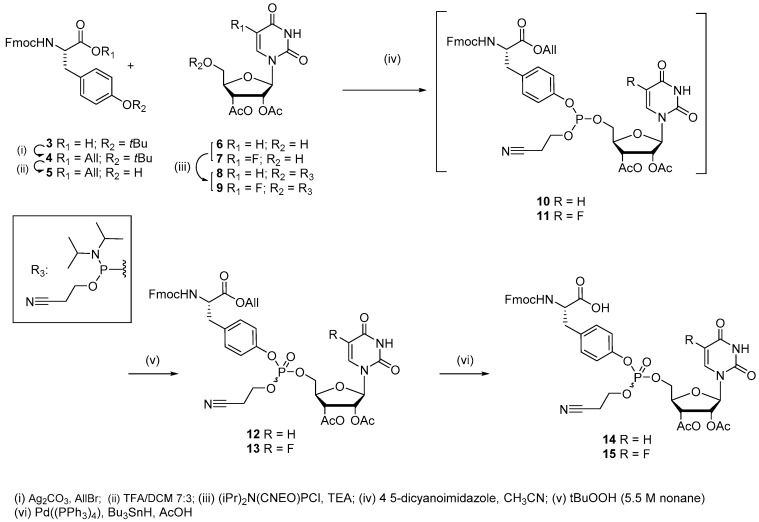
Synthesis of (fluoro)uridylylated tyrosine building blocks.

UMP-VPg1 and FUMP-VPg1 were synthesized following the approach developed by Filippov for the preparation of a poliovirus 22 aa long UMP-VPg using sequential solid-phase peptide synthesis (SPPS) and a pre-uridylylated tyrosine building block [23,24]. We carried out the Fmoc-based SPPS on a Wang resin using the conveniently protected uridylylated or fluorouridylylated tyrosine building blocks **14** and **15** (Scheme 1).

The synthesis started with the preparation of the pre-uridylylated and pre-fluorouridylylated compounds **12** and **13**. Thus, treatment of the Fmoc-Tyr(tBu)-OH **3** with allyl bromide in the presence of Ag_2_CO_3_ gave compound **4** (98% yield) and removal of the tBu Tyr side chain (TFA:CH_2_Cl_2_, 7:3) gave the intermediate **5** in 79% yield (Scheme 1). On the other hand, (fluoro)uridine-5′-phosphoramidites **8** and **9** were prepared upon treatment of corresponding conveniently protected uridine or fluorouridine nucleosides (**6** or **7**) [25,26] with 2-cyanoethyl-*N*,*N*-diisopropylchlorophosphoramidite in the presence of triethylamine [27]. Compounds **8** and **9** were used in the next step without further purification. Thus, coupling of phosphoramidites **8** or **9** with **5** in the presence of 4,5-dicyanoimidazole gave the phosphites **10** and **11,** which, after in situ oxidation with tBuOOH 5.5 M in nonane for 45 min, yielded the fully protected (fluoro)uridylylated tyrosine derivatives **12** and **13**. Finally, palladium(0)-catalyzed hydrostannolysis (Pd((PPh_3_)_4_), Bu_3_SnH, AcOH) of protected compounds **12** and **13** gave the target building blocks **14** and **15** in overall yields of 37% and 28% from **6** or **7**, respectively (Scheme 1).

**Scheme 2:**
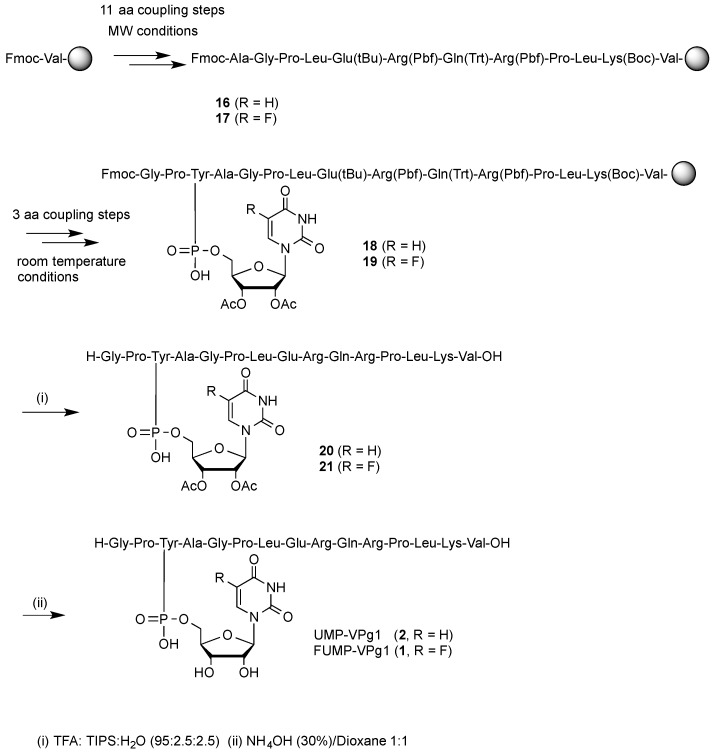
Solid-phase synthesis of FUMP-Vpg1 and UMP-VPg1 models.

Next, the syntheses of the protected nucleopeptides **18** and **19** were performed manually, following standard SPPS protocols, using a Wang polystyrene (1% DVB) resin preloaded with Fmoc-Val-OH and *N*-Fmoc/tBu chemistry (Scheme 2). A low load of the resin (0.4 mmol/g) was used to minimize peptide aggregation and unwanted intermolecular side reactions. Thus, after swelling of the resin, removal of the Fmoc protecting group of the Fmoc-Val residue, under basic conditions (DBU/piperidine/DMF) at room temperature, and sequential coupling of the following eleven amino acids was carried out under microwave irradiation at 40 °C for 10 min, in the presence of HCTU as the coupling reagent and DIEA as the base. A triple coupling protocol (3 × 10 min) was used to give the immobilized 12-mer peptides **16** and **17**. Then, coupling of the next amino acid, the uridylylated or fluorouridylylated tyrosine building blocks (**14** and **15**), was performed in the presence of HCTU/DIEA, following a double coupling protocol (2 × 60 min) at room temperature, to prevent the rupture of the O–P bond (Phosphate bond) and the loss of the nucleotide of **14** or **15**.

The basic conditions required for the elimination of the Fmoc group (DBU/piperidine/DMF) also caused the concomitant elimination of the cyanoethyl group. However, the presence of the phosphodiester group did not interfere in the coupling of the last two amino acids that were also performed at room temperature, also following a double coupling protocol (no unwanted side reactions were detected), to yield the immobilized protected 15-mer peptides **18** and **19**. It has been reported in the literature that the presence of the phosphodiester function does not lead to unwanted by-products [28]. Once elongation of the peptide was completed, removal of the Fmoc group of the *N*-terminus of the nucleopeptides followed by a standard TFA cleavage (TFA/TIPS/water) led to the simultaneous deprotection of the acid labile side-chains protecting group residues and release of the nucleopeptides from the solid support, to give the partially protected nucleopeptides **20** and **21**. The final treatment of acetylated nucleopeptides **20** and **21** with NH_4_OH in dioxane (to deprotect the acetyl groups) yielded the crude target compounds **1** and **2** that were purified by semipreparative HPLC to give pure Vpg1 model compounds **1** and **2** in 18% and 10% global yields, respectively, with a purity of 97% and 94%, respectively (HPLC).

### 2.2. Crystal Structures of the UMP-VPg1 and FUMP-VPg1 Bound to the 3D Polymerase

Previous studies with FMDV 3D^pol^ and the pyrimidine analogue 5-fluorouridine triphosphate (FUTP) revealed that this nucleotide analogue acts in two different ways during FMDV replication: (i) as a mutagenic agent when it is incorporated into the nascent RNA by increasing misincorporation of GMP in place of UMP, and (ii) as a strong inhibitor of VPg uridylylation [18]. Despite the fact that FUMP-VPg1 was observed, this fluorouridylylated form of VPg prevented the incorporation of the second UMP molecule [18]. 

To gain insight into the role of FUMP-VPg1 in inhibition of further uridylylation, we co-crystallized 3D^pol^ in the presence of the modified 15-mer peptides FUMP-VPg1 and UMP-VPg1. Tetragonal crystals of the 3D^pol^ complexes were obtained in both cases. X-ray diffraction data from both crystals were collected at 100 K using synchrotron radiation. The structures were solved by direct molecular replacement, using the tetragonal structure of the unbound polymerase (PDB id. 1U09) as a model. Direct rigid body refinement was sufficient to position the 3D^pol^ model in the new unit cells (Table 1). 

**Figure 3:**
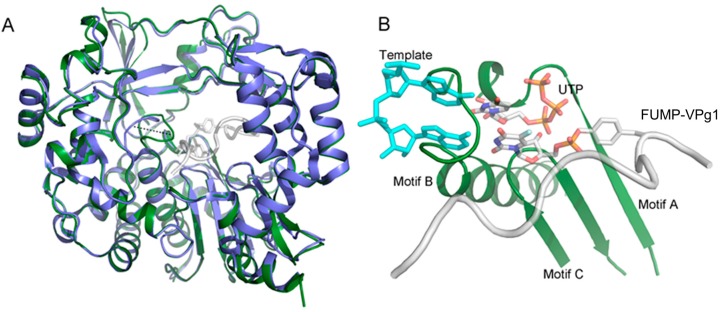
(**A**) The structures of FMDV 3D^pol^ (green) crystallized in presence of the fluorouridylylated VPg that was disordered in the electron density. The structure of the 3D^pol^ VPg1 uridylylated previously has been superimposed to illustrate the conformational diferences (3D^pol^ is in blue and the UMP-VPg1 molecule in white; PDB id 2F8E) The conformational rearrangement of the β9-α11 loop has been highlighted with a dotted line. (**B**) Close-up of the polymerase catalytic cavity in the 3D^pol^ FUMP-VPg1 crystals. The active site motifs A, B (where the β9-α11 loop is located) and C are shown as a green cartoon. FUMP-VPg1 (white C atoms) has been modeled within the cavity with the side chain of Tyr3 covalently attached to FUMP depicted as sticks (the fluorine atom is colored in light cyan). A dinucleotide ApA template (cyan sticks) and an incoming UTP molecule (sticks with C atoms in white) have also been modeled to show the steric clashes that would take place between the template nucleotides and the β9-α11 loop.

Analyses of the electron density maps showed that the VPg1 peptides could not be traced in any of the complexes, most likely due to disorder. However, best-fit structural superpositioning showed that 3D^pol^ crystallized in the presence of FUMP-VPg1 exhibited a large conformational change in the β9-α11 loop, located at the N-terminal motif B. On the contrary, the structure of 3D^pol^ in complex with UMP-VPg1 was practically identical to that of the apo form (Figure 3A). Motif B is formed by a “loop-α-helix” structure located at the base of the template channel and is involved in ribonucleoside triphosphate (rNTP) recognition, template binding and translocation of the nascent dsRNA [29,30,31,32,33]. In the apo form of 3D^pol^, the β9-α11 loop is packed against the fingers domain, forming part of the template channel and participating in template binding. But in the presence of FUMP-VPg1, loop residues from Ser298 to Thr303 in this motif B appear displaced by 7.8 Å towards the active site, thereby closing the template channel and preventing the correct positioning of the nucleotide template in the active site pocket (Figure 3B).

Different studies have shown that the motif B loop is one of the most flexible elements in the active sites of RdRps (reviewed in [31]). Large movements of this loop have been observed ranging from an open conformation, in which the loop is packed against the fingers domain, leaving the active site cavity accessible for template entry, to a closed conformation where the loop is found inside the catalytic pocket, resulting in a conformation that sterically clashes with the template nucleotide (Figure 3B). The structural data shown in this work, together with previous evidence of VPg fluorouridylylation [18], prompt us to speculate that the inhibitory effect of FU on VPg uridylylation might be explained by the stabilization of the β9-α11 loop in the closed conformation, thus preventing the correct positioning of the template nucleotide in the active site cavity of 3D^pol^. Because FUMP-VPg1 is detected experimentally [18] this inhibition would occur following attachment of the first FU unit to VPg. 

### 2.3. Molecular Modeling Studies

**Figure 4:**
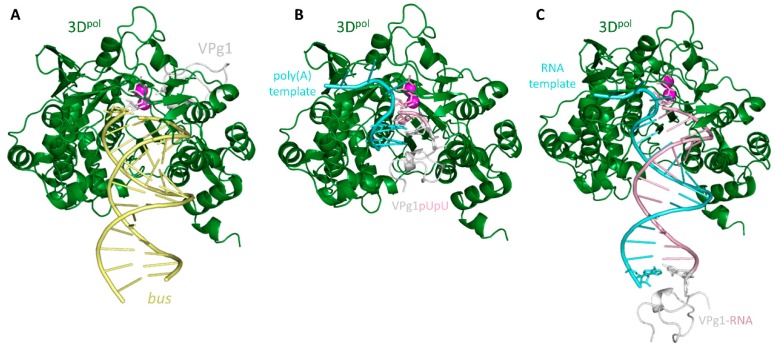
(**A**) Model of FMDV 3D^pol^ (green cartoon) in complex with the bus template (yellow); VPg1 (grey cartoon) sits at the entrance to the active site with the hydroxyl of Tyr3 poised for attack to the α-phosphate of UTP (white sticks). (**B**) Model of FMDV 3D^pol^ in complex with VPg1pUpU paired to the poly(A) template (cyan) and an incoming Mg^2+^-bound UTP. (**C**) Model of replicative FMDV 3D^pol^ in complex with an elongating RNA strand (pink) using the remaining genomic RNA (cyan) as a template. The Mg^2+^ ions in the polymerase active site are displayed as spheres colored in magenta.

The x-ray crystallographic work was completed with some additional model building because, although a second VPg-binding site on picornavirus 3D^pol^ enzymes has been demonstrated [7], one would expect each of the three distinct VPgs of FMDV (i.e., 3B1, 3B2 and 3B3) to bind into the active site of the enzyme in such a way as to make uridylylation possible. This reaction takes place by in-line attack of the Tyr3 hydroxyl of VPg onto the α-phosphorus atom of UTP with the aid of the conserved triad of catalytic aspartates and the bound Mg^2+^ ions in 3D^pol^ [34]. The 3B-uridylylation site (“bus”), located in a hairpin within the 5′-untranslated region of the FMDV genome [35], was predicted by the Mfold [36] and RNAcomposer [37] servers to consist of a base-paired stem and a loop region of sequence C**AA**ACACGAUCUA in which the underlined AAACA motif is strictly conserved. The two contiguous adenines shown in bold (**A**_2_ and **A**_3_) can be considered the putative templates for the addition to VPg peptides of the first and second UMP moieties (Figure 4A), in accordance with similar findings reported for poliovirus [7]. Nonetheless, the proposed “slide-back” mechanism [38], which is similar to that originally proposed for the initiation of protein-primed DNA synthesis by the DNA polymerase of phage ϕ29 [6], may imply that only A_2_ fulfils this role whereas the presence of both A_2_ and A_3_ ensures that the only dinucleotide produced will be VPgpUpU.

From a structural standpoint, VPg1–3 are predicted to be highly disordered peptides but, in order to place the Tyr3 hydroxyl close to the α-phosphorus atom of the UTP molecule and the Mg^2+^-bound Asp240, Asp245 and Asp338, the only entry site possible is between motif A and motif F (Figure 4A). Once Tyr3 becomes uridylylated, a second UTP nucleotide is recruited and a second uridylylation takes place. Since further elongation of VPg1pUpU by 3D^pol^ using bus as the template is aborted [38], there must be a conformational change leading to the release of bus. Thereafter, the resulting 3D^pol^:VPg1pUpU complex readily accepts the 3′-end of the poly(A) tail to be used as the primer for minus strand RNA synthesis. A model of this complex in the presence of the incoming UTP and Mg^2+^ ions is shown in Figure 4B. The intrinsically disordered character of VPg1 is in consonance with the fact that this peptide chain has to slide down the polymerase channel during RNA strand elongation in the 5′→ 3′ direction (Figure 4C). 

Based on our models, we believe that the FUMP moiety is closer to Cys300 in the β9-α11 loop when the active site is occupied by FUMP-VPg in the second uridylylation step than when it is occupied, in the first uridylylation step, by VPg and UTP in the presence of the template RNA hairpin (cre or bus). Our hypothesis would be that the side-chain thiol of Cys300 can attack C6 on the FU ring to give rise to a covalent adduct that causes loop displacement as observed in the electron density map, despite the fact that the excessive disorder of the attached peptide in this large cavity prevents further structural characterization. In this light, the action of FUMP-VPg on FMDV 3D^pol^ would be reminiscent of that of the suicide inhibitor FdUMP—intracellularly generated from the anticancer prodrug FU—forming a covalent complex with an active-site Cys of human thymidylate synthase [39]. Further work is ongoing to test this hypothesis.

## 3. Materials and Methods

### 3.1. Chemistry 

#### 3.1.1. General Methods 

Unless otherwise stated, analytical grade solvents and commercially available reagents were used without further purification. DIEA, piperidine, Ac_2_O, and EDT were purchased from Aldrich (Taufkirchen, Germany), TFA from Fluka (Taufkirchen, Germany) and HCTU from Fluorochem (Derbyshire, UK). Fmoc-protected amino acids were purchased from Fluorochem (Derbyshire, UK), Polypeptide (Strasbourg, France), Novabiochem (Merck, Darmstadt, Germany) and Iris Biotech (Marktredwitz, Germany). THF was distilled over P_2_O_5_; MeCN and DCM were distilled over CaH_2_ prior to use. Solvents were removed by rotary evaporation under reduced pressure at a temperature not exceeding 40 °C.

Pre-loaded Fmoc-Val-Wang resin (0.4 mmol/g) was purchased from Novabiochem. The coupling reactions were carried out on solid phase using microwave radiation in a Biotage Initiator reactor in a 5 mL vial, or at room temperature. Excluding the coupling reactions on the microwave reactor, the rest of the SPPS reactions were stirred using an IKA-100 orbital shaker. After cleavage, the acidic crudes were sedimented in Et_2_O on a centrifuge at 2370 rcf. All the crude and samples were lyophilized using water/acetonitrile mixtures on a Telstar 6–80 instrument. The monitoring of the reactions was performed by HPLC/MS through a HPLC-waters 12,695 connected to a Waters Micromass ZQ spectrometer. Nucleopeptides were purified on a semipreparative HPLC instrument. As mobile phase, mixtures of A:B were used, where A = 0.05% TFA water and B = acetonitrile with a flow rate of 7 mL/min. The purity of the peptides was checked by analytical RP-HPLC on an Agilent Infinity instrument equipped with a Diode Array and a C18 Sunfire column (4.6 mm × 150 mm, 3.5 μm). The mobile phases consisted of: System A, linear gradient solvent system of CH_3_CN/0.05% TFA water from 50:50 to 100:0 in 10 min, flow rate 1.0 mL/min; System B, linear gradient solvent system of CH_3_CN/0.05% TFA water from 10:90 to 100:0 in 10 min, flow rate 1.0 mL/min. HRMS (EI+) was carried out in an Agilent 6520 Accurate-Mass Q-TOF LC/MS spectrometer using water/acetonitrile mixtures.

#### 3.1.2. Synthesis of *Nα*-Fmoc-(*O*-*tert*-butyl)-tyrosine allyl ester (4)

To a solution of *N*α-Fmoc-(*O*-*tert*-butyl)-tyrosine (10 g, 22 mmol) in dry DMF (100 mL) at 0 °C was added Ag_2_CO_3_ (12 g, 44 mmol) under exclusion of light. The reaction mixture was warmed to room temperature and stirred for 15 min prior to addition of allylbromide (12 g, 44 mmol). The mixture was stirred for 2.5 h and then filtered through celite. The filtrate was diluted in 100 mL EtOAc. The organic phase was subsequently washed with 10% KHSO_4_ (2 × 25 mL) and water (2 × 30 mL), dried (MgSO_4_) and concentrated. The residue was applied to a silica gel column (Hexane:EtOAc, 7:3) to yield 10.8 g (98%) of **4**. HPLC (system A): 8.14 min (95% analytical purity). ^1^H-NMR (400 MHz, CDCl_3_) δ: 1.32 (s, 9 H, t-But), 3.09 (m, 2 H, CH_2_ β), 4.21 (t, 1 H, *J* = 7.1 Hz, CH Fmoc), 4.35 (dd, 1 H, *J* = 10.6, 6.9 Hz, CH_2a_ Fmoc), 4.43 (dd, 1 H, J = 10.6, 7.3 Hz, CH_2b_ Fmoc), 4.63 (m, 3 H, CH_2_ All, CH α), 5.28 (m, 3 H, CH_2_ All, NH), 5.86 (m, 1 H, CH All), 6.90 (d, 2 H, *J* = 8.3 Hz, Ar Tyr), 7.01 (d, 2 H, *J* = 8.3 Hz, Ar Tyr), 7.32 (t, 2 H, *J* = 7.4 Hz, Ar Fmoc), 7.41 (t, 2 H, *J* = 7.4 Hz, Ar Fmoc), 7.57 (m, 2 H, Ar Fmoc), 7.76 (d, 2 H, *J* = 7.5 Hz, Ar Fmoc). HRMS: [C_31_H_33_NO_5_]^+^; calcd. 499.2350, found 499.2359.

#### 3.1.3. Synthesis of *Nα*-Fmoc-tyrosine-allyl ester (5)

A solution of allyl ester **4** (10.8 g, 22 mmol) in a mixture TFA:DCM (7:3, *v*/*v*) was stirred for 30 min at room temperature. Toluene (50 mL) was added and the solvents were removed. The residue was purified on a silica gel column (toluene:EtOAc, 9:1) to yield 7.7 g (79%) of **5**. HPLC (system A): 5.09 min (93% analytical purity). ^1^H-NMR (400 MHz, CDCl_3_) δ: 3.02 (dd, 1 H, *J* = 14.0, 6.1 Hz, CH_2a_ β), 3.09 (dd, 1 H, *J* = 14.0 y 5.6 Hz, CH_2b_ β), 4.20 (t, 1 H, *J* = 7.0 Hz, CH Fmoc), 4.36 (dd, 1 H, *J* = 10.6, 6.9 Hz, CH_2a_ Fmoc), 4.43 (dd, 1 H, *J* = 10.6 y 7.3 Hz, CH_2b_ Fmoc), 4,.66 (m, 3 H, CH_2_ All, CH α), 5.28 (m, 3 H, CH_2_ All, NH), 5.88 (m, 1 H, CH All), 6.72 (d, 2 H, *J* = 8.3 Hz, Ar Tyr), 6.95 (d, 2 H, *J* = 8.3 Hz, Ar Tyr), 7.31 (t, 2 H, *J* = 7.4 Hz, Ar Fmoc), 7.40 (t, 2 H, *J* = 7.4 Hz, Ar Fmoc), 7.57 (m, 2 H, Ar Fmoc), 7.76 (d, 2 H, *J* = 7.5 Hz, Ar Fmoc). HRMS: [C_27_H_25_NO_5_]^+^; calcd. 443.1736, found 443.1733. α_D_ (CHCl_3_) = +14.6°. HRMS. α_D_ (CHCl_3_) = +21.3°, α_D_ (CHCl_3_)^12^ = +16.8°.

#### 3.1.4. Synthesis of 2-cyanoethyl-(Nα-Fmoc-tyrosin-4-yl-allylester)-(2′,3′-di-*O*-acetyluridyl-5′-yl)phosphate (12)

Phosphoramidite **8** was prepared by reacting 2′,3′-di-*O*-acetyluridine (1.15 g, 3.52 mmol), coevaporated with MeCN (2 × 5 mL) in DCM (15 mL), containing TEA (2.25 mL, 16.70 mmol), with 2-cyanoethoxy-*N,N*-diisopropylaminochlorophosphine (0.95 mL, 4.22 mmol) under an argon atmosphere. The reaction mixture was stirred for 30 min at room temperature. DCM (20 mL) was added and the reaction mixture was washed with water (20 mL) and with 5% NaHCO_3_ (2 × 20 mL). The organic phase was dried (MgSO_4_) and concentrated to yield **8**, which was used in the next step without further purification. HPLC (system B): 4.68 min (96% analytical purity). Approximately 1 mmol of **8** was added to a solution of *N*α-Fmoc-tyrosine-allyl ester (**5**) (443 mg, 1 mmol) in MeCN (10 mL). 4,5-Dicyanoimidazole (177 mg, 1.5 mmol) was added and the reaction mixture was stirred for 45 min. A solution of tBuOOH (5.5 M in nonane, 0.73 mL, 4 mmol) was added to the reaction mixture and stirred for 45 additional minutes. The reaction mixture was diluted in 25 mL DCM and washed with 5% Na_2_S_2_O_3_ (3 × 20 mL) and water (20 mL). The organic phase was dried (MgSO_4_). The solvent was removed under reduced pressure, and the residue was purified by flash column chromatography (DCM:MeOH, 98:2) to obtain 505 mg (57%) of **12**. HPLC (system B): 8.85 min (91% analytical purity).^1^H-NMR (400 MHz, DMSO-d_6_) δ: 2.06 (s, 6 H, COCH_3_), 2.85 (m, 1 H, CH_2a_ β), 2.94 (t, 2 H, *J* = 5.8 Hz, CH_2_CN), 3.06 (m, 1 H, CH_2b_ β), 4.20 (t, 1 H, *J* = 7 Hz, CH Fmoc), 4.25–4.71 (m, 10 H, CH_2_ Fmoc, CH_2_ All, CH_2_CH_2_CN, CH_α_, H-4′, H-5′), 5.12–5.37 (m, 3 H, H-2′, CH_2_ All), 5.37–5.45 (m, 1 H, H-3′), 5.58–5.79 (m, 1 H, H-5), 5.82–5.96 (m, 1 H, CH All), 6.00–6.13 (m, 1 H, H-1‘), 7.04–7.20 (m, 4 H, Ar Tyr), 7.26–7.34 (m, 2 H, Ar Fmoc), 7.35–7.45 (m, 3 H, Ar Fmoc, H-6), 7.53–7.62 (m, 2 H, Ar Fmoc), 7.76 (m, 2 H, Ar Fmoc). ^31^P-NMR (162 MHz, DMSO-d_6_) δ: –6.18, –5.82. HRMS: [C_43_H_43_N_4_O_15_P]^+^; calcd. 886.2463, found 886.2463.

#### 3.1.5. Synthesis of 2-cyanoethyl-(*Nα*-Fmoc-tyrosin-4-yl-allylester)-(2′,3′-di-*O*-acetyl-5-flurouridyl-5′-yl) phosphate (13)

Phosphoramidite **9** was prepared by reacting 2′,3′-di-*O*-acetyl-5-fluorouridine (1.21 g, 3.5 mmol), coevaporated with MeCN (2 × 5 mL) in DCM (15 mL), containing TEA (2.25 mL, 16.70 mmol), with 2-cyanoethoxy-*N,N*-diisopropylaminochlorophosphine (0.95 mL, 4.22 mmol) under an argon atmosphere. The reaction mixture was stirred for 30 min at room temperature. DCM (20 mL) was added and the reaction mixture was washed with water (20 mL) and 5% NaHCO_3_ (2 × 20 mL). The organic phase was dried (MgSO_4_) and concentrated. HPLC (system B): 4.68 min (96% analytical purity). Approximately 1 mmol of **9** was added to a solution of *N*α-Fmoc-tyrosine-allyl ester (**5**) (443 mg, 1 mmol) in MeCN (10 mL), then 4,5-dicyanoimidazole (177 mg, 1.5 mmol) was added and the reaction mixture was stirred for 45 min. A solution of tBuOOH (5.5 M in nonane, 0.73 mL, 4 mmol) was added to the reaction mixture and stirred for 45 additional minutes. The reaction mixture was diluted in 25 mL DCM and washed with 5% Na_2_S_2_O_3_ (3 × 20 mL) and water (20 mL). The organic phase was dried (MgSO4). The solvent was removed under reduced pressure, and the residue was purified by flash column chromatography (DCM:MeOH, 98:2) to obtain 858 mg (42%) of **13**. HPLC (system B): 8.91 min (99% analytical purity). ^1^H-NMR (400 MHz, DMSO-d_6_) δ: 2.05, 2.07 (s, 6 H, COCH_3_), 2.94 (m, 3 H, CH_2a_ β, CH_2_CN), 3.05 (dd, 1 H, *J* = 13.7, 5.1 Hz, CH_2b_ β), 4.17–4.34 (m, 7 H, CH_2_ Fmoc, CH Fmoc, CH α, CH_2_CH_2_CN, H-4′), 4.43 (m, 2 H, H-5′), 4.57 (m, 2 H, CH_2_ All), 5.18 (dd, 1 H, *J* = 10.6, 1.6 Hz, CH_2a_ All), 5.28 (dd, 1 H, *J* = 17.2, 1.6 Hz, CH_2b_ All), 5.34 (m, 1H, H-3′), 5.43 (m, 1 H, H-2′), 5.84 (m, 1 H, CH All), 5.93 (d, 1 H, *J* = 5.7 Hz, H-1′), 7.15 (d, 2 H, *J* = 8.3 Hz, Ar Tyr), 7.26–7.35 (m, 4 H, Ar Fmoc y Tyr), 7.41 (t, 2 H, *J* = 7.5 Hz, Ar Fmoc), 7.65 (m, 2 H, Ar Fmoc), 7.88 (d, 2 H, *J* = 7.5 Hz, Ar Fmoc), 7.92 (bs, 1H, NH), 8.09 (m, 1H, H-6), 12.0 (bs, 1H, NH). ^13^C-NMR (100 MHz, DMSO-d_6_) δ: 19.0, 19.1 (CH_2_CN), 20.2, 20.3 (COCH_3_), 35.6 (CH_2_ β), 46.6 (CH Fmoc), 55.5 (CH α), 63.3 (CH_2_CH_2_CN), 64.9 (CH_2_ All), 65.6 (CH_2_, Fmoc), 67.0 (C-5′), 69.0 (C-3′), 71.5 (C-2′), 79.6 (C-4′), 87.4 (C-1′), 117.8 (CH_2_ All), 118.1 (CN), 119.7 (CH Ar Tyr), 120.1 (CH Ar Fmoc), 125.3 (CH Ar Fmoc), 125.2 (C-6, *^2^J* = 36 Hz), 127.1, 127.7 (CH Ar Fmoc), 130.6 (CH Ar Tyr), 132.2 (CH All), 134.7 (C Tyr), 139.6 (C-5, *^1^J* = 232.1 Hz), 140.7 (C Fmoc), 143.7 (C Fmoc), 148.6 (C Tyr), 149.1 (C=O C-2), 155.9 (CONH), 156.9 (C=O C-4, *^2^J* = 26.2 Hz), 169.3 (COCH_3_), 173.3 (C=O Tyr). ^31^P-NMR (162 MHz, DMSO-d_6_) δ: –5.83, –5.87. HRMS: [C_43_H_42_FN_4_O_15_P]^+^; calcd. 904.2401, found 904.2402.

#### 3.1.6. Synthesis of 2-cyanoethyl-(*Nα*-Fmoc-tyrosin-4-yl)-(2′,3′-di-*O*-acetyluridyl-5′-yl)phosphate (14)

To a stirred solution of **12** (1.6 g, 1.8 mmol), coevaporated with MeCN (5 mL × 2) in 1:1 THF/DCM (10 mL, *v*/*v*), containing AcOH (0.45 mL, 8 mmol), Bu_3_SnH (0.97 mL, 3.6 mmol) and Pd(PPh_3_)_4_ (87 mg, 0.08 mmol) were added under an argon atmosphere. After two hours the reaction mixture was concentrated and purified by flash column chromatography (DCM:MeOH, 94:6) to yield **14** (0.99 g, 65%) as white amorphous solid. HPLC (system B): 7.63 min (97% analytical purity). ^1^H-NMR (400 MHz, DMSO-d_6_) δ: 2.06 (s, 6 H, COCH_3_), 2.85 (m, 1 H, CH_2a_ β), 2.94 (t, 2 H, *J* = 5.8 Hz, CH_2_CN), 3.06 (m, 1 H, CH_2b_ β), 4.18 (m, 4 H, CH_2_ Fmoc, CH Fmoc, CH_α_), 4.30 (m, 3 H, CH_2_CH_2_CN, H-4′), 4.41 (m, 2 H, H-5′), 5.34 (m, 1 H, H-3′), 5.44 (m, 1 H, H-2′), 5.67 (m, 1 H, H-5), 5.93 (d, 1 H, *J* = 5.6 Hz, H-1′), 7.11–7.92 (m, 13 H, Ar Fmoc y Tyr, H-6), 11.4 (bs, 1 H, NH). ^31^P-NMR (162 MHz, DMSO-d_6_) δ: –11.10, –10.65. HRMS: [C_40_H_39_N_4_O_15_P]^+^; calcd. 846.2149, found 846.2150.

#### 3.1.7. Synthesis of 2-cyanoethyl-(*Nα*-Fmoc-tyrosin-4-yl)-(2′,3′-di-*O*-acetyl-5-fluorouridyl-5′-yl) phosphate (15)

To a stirred solution of **13** (1.52 g, 1.68 mmol), coevaporated with MeCN (5 mL × 2) in a 1:1 mixture of THF/DCM (10 mL, *v*/*v*), containing AcOH (0.42 mL, 8 mmol), Bu_3_SnH (0.91 mL, 3.36 mmol) and Pd(PPh_3_)_4_ (81 mg, 0.07 mmol) were added under an argon atmosphere. After two hours the reaction mixture was concentrated and purified by flash column chromatography (DCM:MeOH, 94:6) to yield **15** (0.97 g, 67%) as white amorphous solid. HPLC (system B): 7.80 min (95% analytical purity). ^1^H-NMR (400 MHz, DMSO-d_6_) δ: 2.06 (s, 6 H, COCH_3_), 2.86 (dd, 1 H, *J* = 13.7, 2.8 Hz, CH_2a_ β), 2.94 (t, 2 H, *J* = 5.8 Hz, CH_2_CN), 3.08 (dd, 1 H, *J* = 13.7, 4.0 Hz, CH_2b_ β), 4.19 (m, 4 H, CH_2_ Fmoc, CH Fmoc, CH α), 4.29 (m, 3 H, CH_2_CH_2_CN, H-4′), 4.43 (m, 2 H, H-5′), 5.34 (m, 1 H, H-3′), 5.44 (m, 1 H, H-2′), 5.93 (d, 1 H, *J* = 5.6 Hz, H-1‘), 7.15 (d, 2 H, *J* = 8.3 Hz, Ar Tyr), 7.29–7.45 (m, 6 H, Ar Fmoc y Tyr), 7.65 (t, 2 H, *J* = 7.3 Hz, Ar Fmoc), 7.75 (bs, 1 H, NH), 7.88 (d, 2 H, *J* = 7.6 Hz, Ar Fmoc), 8.09 (t, 1 H, *J* = 6.6 Hz, H-6), 12.0 (bs, 1 H, NH). ^13^C-NMR (100 MHz, DMSO-d_6_) δ: 19.0, 19.1 (CH_2_CN), 20.3, 20.4 (COCH_3_), 35.6 (CH_2_ β), 46.6 (CH Fmoc), 55.5 (CH α), 63.3 (CH_2_CH_2_CN), 65.6 (CH_2_ Fmoc), 67.0 (C-5′), 69.0 (C-3′), 71.5 (C-2′), 79.6 (C-4′), 87.4 (C-1′), 118.1 (CN), 119.7 (CH Ar, Tyr), 120.1 (CH Ar, Fmoc), 125.3 (CH Ar, Fmoc), 125.6 (C-6, *^2^J* = 36 Hz), 127.1, 127.7 (CH Ar, Fmoc), 130.6 (CH Ar, Tyr), 135.4 (C Tyr), 139.6 (C-5, *^1^J* = 234.7 Hz), 140.7 (C Fmoc), 143.8 (C Fmoc), 148.5 (C Tyr), 149.1 (C=O C-2), 156.0 (CONH), 157.0 (C=O C-4, *^2^J* = 26.3 Hz), 169.4 (COCH_3_), 173.3 (C=O Tyr). ^31^P-NMR (162 MHz, DMSO-d_6_) δ: –11.50, –11.40. HRMS: [C_40_H_38_FN_4_O_15_P]^+^; calcd. 864.2047, found 864.2055.

#### 3.1.8. General Procedure for the SPPS of Nucleopeptides. 

Nucleopeptides **1, 2** were synthesized manually on resin following the standard Fmoc/tBu solid phase orthogonal protection strategy. Preload Fmoc-Val-Wang resin (0.4 mmol) was swollen DCM/DMF/DCM/DMF (4 × 0.5 min), then the resin was treated with a mixture of DBU:piperidine:DMF (1:1:48, in volume) at room temperature (1 × 1 min) and (3 × 10 min) and washed with DMF/DCM/DMF/DCM (4 × 0.5 min). Later, to the free Nα-terminal swollen resin (1 equiv), a solution of the corresponding Fmoc-AA-OH (1.2 equiv.), HCTU (1.2 equiv.) and DIEA (2.4 equiv.) in dry DMF (5 mL) was added. 

The introduction of the first 12 amino acids was carried out under microwave radiation while the introduction of the last three amino acids was carried out at room temperature to prevent the rupture of the phosphate bond.

(1) Coupling procedure of the first 11 amino acids: After sealing the vial, the reaction was heated in a microwave vial equipped with a magnetic stirrer for 10 min at 40 °C. Then, the vial was opened, the supernatant removed and new coupling mixture added. This process was repeated three times in total (3 × 10 min) until complete coupling. Finally, the resin was transferred to a fritted syringe, drained and washed extensively (DMF/DCM/DMF/DCM, 5 × 0.5 min). This protocol was repeated for the sequential anchoring of each of the first 12 amino acids.
(2) Coupling of the last three amino acids: The mixture was stirred 1 h at room temperature. Then, the vial was opened, the supernatant removed and new coupling mixture added. Finally, the resin was drained and washed extensively (DMF/DCM/DMF/DCM, 5 × 0.5 min). This protocol was repeated for the sequential anchoring of each of the last three amino acids of the sequence.

Coupling reactions to primary amines were monitored by the Kaiser ninhydrin test and to secondary amines by the chloranil test. In some cases, the progress of the reactions was also followed by analysis of a small sample of peptidylresin after acidic cleavage in an HPLC-MS instrument. After elongation of the peptides, the well dried resin-bounded derivative (1 vol) in a fritted syringe was treated with a mixture of TFA:TIPS:water (95:2.5:2.5) (5 vol) for 4 h at room temperature. The filtrates were precipitated over cold Et_2_O and centrifuged three times at 5000 rpm for 10 min. After removing the supernatant, the pellet was redissolved in water/acetonitrile and lyophilized. The crudes were purified by reverse phase chromatography using semipreparative HPLC.

H-Gly-Pro-Tyr(pFU)-Ala-Gly-Pro-Leu-Glu-Arg-Gln-Arg-Pro-Leu-Lys-Val-OH (**1**)

The general protocol was followed with 0.039 mmol of resin. After purification of the crude, **1** was isolated as a white lyophilized cotton-like solid (13 mg, 10% overall yield). HPLC (system B): 4.73 min (94% analytical purity). HRMS: [C_85_H_135_FN_25_O_28_P]^+^; calcd. 2003.9611, found 2003.9630.

H-Gly-Pro-Tyr(pU)-Ala-Gly-Pro-Leu-Glu-Arg-Gln-Arg-Pro-Leu-Lys-Val-OH (**2**)

The general protocol was followed with 0.039 mmol of resin. After purification of the crude, **2** was isolated as a white lyophilized cotton-like solid (14 mg, 18% overall yield). HPLC (system B): 4.43 min (97% analytical purity). HRMS: [C_85_H_136_N_25_O_28_P]^+^; calcd. 1985.9746, found. 1985.9724. 

### 3.2. Crystallization, Data Collection and Processing

To analyze the effect of uridylylated and fluorouridylylated VPgs (UMP-VPg1 and FUMP-VPg1, respectively) on the polymerase activity, 3D^pol^ was incubated overnight in presence of equimolar proportions of a UMP-VPg1 and FUMP-VPg1. Crystals were obtained by the hanging-drop vapor-diffusion method at 20 °C in a 24 well Greiner plate, crystallization drops contained 1 μL of protein solution and 1 μL of crystallization buffer (0.2 M ammonium acetate, 30% PEG 4 K, 0.1 M MES pH 6.0 and 4% γ-butyrolactone) and were equilibrated against 1 mL of crystallization buffer. Crystals appeared in 2 or 3 days. Prior to data collection, crystals were transferred to a cryo-protecting solution in a reservoir solution and 20% (*v*/*v*) glycerol, and flash-frozen in liquid nitrogen. 

Crystals that contain FUMP-VPg1 were collected at 100 K at the ESRF beamline ID29 (Grenoble, France). Diffraction images were processed with XDS [40] and internally scaled with SCALA [41]. Best crystals, belonging to the tetragonal space group P4_1_2_1_2_1_ diffracted to 2.3 Å resolution (Table 1). The structure of this complex was determined by following a rigid body refinement procedure with Refmac5 [42], using the structure of the unbound FMDV 3D^pol^ (PDB id.1U09) as an initial model. This model was manually rebuilt, using Coot [43] and positionally refined with Refmac5 [42] and Phenix [44]. The statistics of the refinement are summarized in Table 1. The refined coordinates and structure factors are available at the PDB under accession code 6S2L. 

### 3.3. Molecular Modeling Methods 

The RNA secondary structure predicted by Mfold [36] and RNAcomposer [37] servers for the bus hairpin was used as input for program ROSIE [45,46], which produced different sets of three-dimensional coordinates. Robetta [47,48] was used for generation of both ab initio and comparative models of VPg1. PyMOL [49] was employed for structure visualization, molecular editing and figure preparation. The nucleic acids and nucleotides docked in the polymerase active site of FMDV 3D^pol^ were modeled by gathering information from the structurally better characterized active site of HIV reverse transcriptase (PDB entries 1RTD and 5XN2) upon best-fit RMSD superimposition. Geometry optimization of the resulting complexes was achieved by carrying out 50,000 steps of steepest descent followed by 50,000 steps of conjugate gradient energy minimization in the AMBER force field [50] using the sander module of AMBER 16. 

## 4. Conclusions

FUTP acts as a potent inhibitor of the second VPg uridylylation step that takes place during initiation of FMDV replication. However, the molecular mechanism of this inhibition is not fully understood. The crystal structure of FMDV 3D^pol^ in complex with the synthetic 15 aa model peptide FUMP-VPg1 reveals a large conformational change of the β9-α11 loop of the polymerase that is likely to block the access of the template strand to the catalytic cavity. The results obtained provide insights into the mechanism of inhibition of this nucleoside analogue and should facilitate the design of new antivirals against FMDV or other picornavirus since VPg uridylylation is a common process in all of them.

## Figures and Tables

**Table 1:** Data collection and refinement statistics.

**Data Collection**	3D^pol^-FUMP-VPg1
Beamline	ID29 (ESRF)
Resolution (Å)	74.23–2.30 (2.42–2.30)
Space Group	P4_1_2_1_2
Cell dimensions	
a, b, c (Å)	93.85 93.85 121.31
α, β, γ (°)	90 90 90
Rmerge	0.092 (0.77)
I/σI	12.7 (2.5)
Completeness (%)	99.9 (100)
Multiplicity	6.6 (6.9)
**Refinement**	
Resolution (Å)	74.23–2.3
No. reflections (total/unique)	162,185/24,747
R_work_ †/R_free_ ‡	22.88/26.91
No. Atoms/Residues	
3D^pol^	3918 (476)
Waters	148
B-factors (Å^2^)	
All atoms	27.41
3D^pol^	27.54
Waters	26.94
R.m.s. deviations	
Bond lengths (Å)	0.006
Bond angles (°)	0.892
Ramachandran plot	
Residues in preferred regions	462 (97.5%)
Residues in allowed regions	10 (2.1%)

† R*_work_* = ∑hkl ||Fobs(hkl)| - |Fcalc(hkl)||/∑hkl |Fobs(hkl)|, where Fobs and Fcalc are the structure factors, deduced from measured intensities and calculated from the model, respectively. ‡ R*_free_* = as for R*_work_* but for 5% of the total reflections chosen at random and omitted from refinement.
